# Prenatal Testosterone Exposure Disrupts Insulin Secretion And Promotes Insulin Resistance

**DOI:** 10.1038/s41598-019-57197-x

**Published:** 2020-01-15

**Authors:** Albert Carrasco, Mónica P. Recabarren, Pedro P. Rojas-García, Mario Gutiérrez, Karina Morales, Teresa Sir-Petermann, Sergio E. Recabarren

**Affiliations:** 1 Laboratory of Animal Physiology and Endocrinology, Faculty of Veterinary Sciences, Universidad de Concepción, Chillán, Chile; 2 Laboratory of Endocrinology and Metabolism, Western Faculty of Medicine, Universidad de Chile, Santiago, Chile

**Keywords:** Type 2 diabetes, Type 2 diabetes

## Abstract

Hyperandrogenemia and metabolic disturbances during postnatal life are strongly linked both to polycystic ovary syndrome and other conditions that arise from prenatal exposure to androgen excess. In an animal model of this condition, we reported that insulin sensitivity (IS) was lower in young female sheep born to testosterone-treated mothers versus sheep born to non-exposed mothers (control). This lower insulin sensitivity remains throughout reproductive life. However, it is unknown whether abnormal postnatal levels of testosterone (T) further decrease IS derived from prenatal exposure to testosterone. Therefore, we assessed the effects of an acute testosterone administration (40 mg) on IS and insulin secretion during an intravenous glucose tolerance test performed at 40 weeks of age (adulthood) in previously ovariectomized sheep at 26 weeks of age (prepuberty), that were either prenatally exposed to testosterone (T-females, n = 6) or not (C-females, n = 6). The incremental area under the curve of insulin was greater in C-females both with or without the acute testosterone treatment (P < 0.05). The ISI-Composite was lower after an acute testosterone treatment, only in T-females. We conclude that prenatal exposure to testosterone disrupts pancreatic insulin secretion in response to glucose and that in this setting further hyperandrogenemia may predispose to lower insulin sensitivity.

## Introduction

Increased plasma testosterone concentrations during pregnancy could provide an anomalous intrauterine environment for a female fetus, resulting in polycystic ovary syndrome (PCOS) during reproductive life^1^. PCOS carries hyperandrogenemia in the majority of cases^2^ but also metabolic disarrangements such as relative hyperinsulinemia and insulin resistance, two features that predispose to cardiovascular diseases^3,4^. Reduced insulin sensitivity promotes the release of more insulin by the pancreas in order to compensate and achieve its biological effects^5^.

The relationship between androgens and insulin in PCOS patients deserves more understanding to comprehend the pathophysiology of the syndrome. Insulin can increase circulating androgens by a complex molecular mechanism involving the insulin receptor substrate 1 (IRS-1) in the ovaries and the adrenal gland^6^. Metformin, an insulin sensitizer, can reduce hyperinsulinemia to normal levels, but it also reduces hyperandrogenemia^7,8^. However, less is known about the effects of testosterone on insulin secretion and whether it is involved in the pathogenesis of the metabolic condition in PCOS. Adult women diagnosed with PCOS have a higher incidence of insulin resistance when their free testosterone concentrations are higher^9^, suggesting a deleterious effect of plasma testosterone concentrations on insulin resistance.

Divergent effects of testosterone on pancreatic tissue have been reported *in vivo* and *in vitro*, making it difficult to predict a precise role for testosterone on insulin secretion. A stimulatory effect of testosterone on insulin secretion has been reported in men, in whom lower testosterone levels are associated with insulin resistance^10^. *In vitro* exposure of pancreatic tissue to testosterone increase insulin synthesis and secretion, whereas gonadectomy decreases both processes in male rats^11^, suggesting a direct effect of androgens on pancreatic beta cell function. In βARKO male mice, which lack the expression of the androgen receptor (AR) in pancreatic beta cells, a decrease in glucose-stimulated insulin secretion has been observed^12^. Furthermore, dihydrotestosterone (DHT) acts as an insulinotropic agent^12^. In addition, it has been postulated that testosterone has a protective role on pancreatic beta cells, perhaps mediated by the androgen receptor^13^. Therefore, testosterone could have supportive or beneficial roles for the secretion of insulin by the pancreas. The offspring of monkey and sheep prenatally exposed to testosterone excess, show programming effects, that culminate in insulin resistance during postnatal life^14,15^. In PCOS animal models the role of testosterone on insulin resistance is not clear.

In order to explore the effect of prenatal androgen exposure and of adult hyperandrogenemia on insulin we studied insulin secretion and sensitivity before and after a single dose of testosterone in ovariectomized ewes, exposed and not exposed prenatally to testosterone.

## Materials and Methods

### General management and group allocation

All procedures, management and experimental methodologies were previously revised and approved for the Ethical Committee in Animal Research of the Faculty of Veterinary Science of the Universidad de Concepción, Chile and were performed in agreement with the International Guiding Principles for Biomedical Research Involving Animals and Bioethics Advisory Committee of the Chilean National Commission for Scientific and Technological Research (CONICYT, Chile).

Adult female Suffolk-Down sheep were randomly selected for estrous synchronization and mated with rams of proven fertility. The day of mating was recorded and after pregnancy was confirmed by ultrasonography, dams were allocated randomly to be or not to be treated. Treated dams received biweekly intramuscular injections of 30 mg of testosterone (Testosterone propionate, Steraloids, USA) dissolved in 1 ml of vegetable oil per animal, from days 30 to 90 of gestation and thereafter biweekly injections with 40 mg of testosterone per animal until day 120 of gestation. Control dams were injected with vehicle of testosterone following the same schedule. Dams had access to pasture and hay, supplemented with pelleted food according to feeding protocols for pregnant sheep. On day 120 of pregnancy blood samples were collected from pregnant dams by venipuncture after an overnight fast and plasma testosterone concentrations were determined afterwards.

Immediately after birth (~147 days), all newborn lambs were weighed and then left undisturbed to facilitate mother-lamb interactions and to ensure colostrum intake. Lambs were kept with their mothers under natural photoperiod and with free access to water and were weaned at 8-weeks of age. Lamb grouping were assigned as T-females for those born to testosterone-treated dams and C-females for those born to control dams. All females were subjected to a bilateral ovariectomy under general anesthesia at 24 weeks of age in order to mitigate the influence of naturally produced androgens derived from the ovary^6^. Sheep did not receive exogenous estradiol or progesterone after ovariectomy.

### Experimental protocol for intravenous glucose tolerance test (IVGTT) and testosterone acute treatment

The experimental protocol was designed to evaluate insulin secretion and insulin sensitivity by means of an IVGTT in adult (40–42 weeks of age) ovariectomized T-females and C-females sheep (~14–16 weeks after ovariectomy). The first IVGTT was performed at 40 weeks of age. There test was performed without the influence of exogenous androgens administration; in order to determine effects of prenatal exposure to testosterone on insulin sensitivity without the influence of testosterone. The second IVGTT was performed at 42 weeks of age, and it was performed 48 hours after an intramuscular injection of 40 mg of testosterone propionate (Steraloids, USA) dissolved in 1 ml vegetable oil. Both C-females and T-females received the treatment. This second test allowed us to determine the effects of plasma testosterone in addition to a prenatal exposure to testosterone on insulin sensitivity and the effect of testosterone per se on insulin sensitivity by comparing each group separately.

For the IVGTT, bilateral jugular vein catheterization was done. For acclimation, three blood samples were collected prior to the study. Sheep were placed in experimented crates the night before the study and fasted overnight (12 hours). The IVGTT consisted of an infusion, over a 2-minutes interval, of a glucose solution (300 mg glucose/kg body weight^0.75^), as described^15^. At 20 minutes after the start of the glucose infusion, an intravenous bolus of human insulin (0.1 IU/kg body weight, Humulin-R EllyLilly, Santiago, Chile) was administered. Blood samples (1.5 ml) were collected at -15–10, 0 (glucose infusion) and 3, 5, 7, 10, 13, 15, 17, 20 (insulin infusion), 23, 25, 27, 30, 33, 35, 37, 40, 50, 60, 70, 80, 90, 100, 110, 120, 130, 140, 150, 160, 170 and 180 minutes after the start of the glucose infusion. Blood samples were placed in two series of tubes. One series contained sodium fluoride 3% and heparin (for glucose) and the others, contained heparin (1UI/ml blood), for insulin determination. Blood samples were centrifuged at 1,000 x g for 10 minutes at 4 °C. Plasma was collected and deposited in microtubes and stored at -20 °C pending quantification of plasma concentrations of glucose and insulin. Plasma testosterone concentration was measured in the time 0 sample.

### Glucose, insulin and testosterone determination

Plasma glucose concentrations were quantified by a commercial kit (Farmalatina, Chile), based on the glucose oxidase and peroxidase reaction, with measurement of the light absorbance at 505 nm wavelength in a spectrophotometer. Plasma insulin concentrations were determined by IRMA (Biosource, Belgium). Minimal detectable insulin concentration was 1 µIU/ml. Intra-and inter-assays coefficients of variation were 2.68% and 2.63%, respectively. Plasma testosterone concentrations were determined by radioimmunoassay with commercial kit (Biosource, Belgium) in one single assay. The minimal detectable level of testosterone was 0.01 ng/ml and the intra-assay coefficient of variation was 3.92%.

### Indices of insulin sensitivity and insulin production in response to glucose infusion

We determined the following three end points: i) Fasting insulin-to-glucose ratio (I/G ratio), using mean concentrations of insulin and glucose in plasma samples obtained at -15, -10, and 0 min of glucose infusion (basal samples); ii) Composite insulin sensitivity index (ISI-C), based on formula developed by Matsuda and De Fronzo^16^, where ISI-C = 10000/square root of ((fasting glucose × fasting insulin) × (mean glucose × mean insulin during the first 20 min of IVGTT)); and iii) Glucose disappearance rate (GDR), where glucose response to the exogenous insulin injection was calculated using the formula described by Grulet *et al*.^17^. Production of insulin by pancreatic β cells was assessed by calculating the incremental area under the curve of insulin (AUC-I), where the difference between basal AUC of insulin and total AUC of insulin during the first 20-min of the test was obtained with the trapezoidal formula using an Excel® spreadsheet. Complementary information on these formulas is described^15^.

### Statistical analysis

Plasma glucose and insulin concentrations during the first 20 minutes of the IVGTT between C- and T-females was compared by means of a two-way ANOVA for repeated measurements, in with prenatal treatment as the main factor and time as the second factor. Tukey´s multiple comparison was used to compare differences within groups. Differences between C-females and T-females, either with or without the acute testosterone treatment, were determined by unpaired Student’s t-test. Differences within groups, before and after the acute testosterone treatment, were determined with a paired Student’s t-test. Non-parametric tests were used when variances were not similar between groups. Body weights at birth and at 24, 40 and 42 weeks age and plasma testosterone were compared between groups with an unpaired Student’s t-test. Statistical analyses were performed with GraphPad Prism® version 6.0 software, in with P < 0.05 considered different. Data are shown as mean ± standard error.

## Results

### Body weight in ovariectomized sheep

Mean body weight was similar between C- females and T- females except at birth, when T-females were significantly heavier than C-females (Table 1).

**Table 1:** Body weights (Kg) of control (C-females) and prenatally T exposed (T-females) ovariectomized sheep at various ages.

Age	C-females (n = 6)	T-females (n = 6)
Birth	3.5 ± 0.16	4.5 ± 0.17*
24 weeks (ovariectomy)	25.5 ± 1.80	24.6 ± 1.20
40 weeks (IVGTT pre acute T^ϕ^)	34.1 ± 2.00	30.8 ± 1.20
42 weeks (IVGTT post acute T^ϕ^)	32.7 ± 1.80	29.8 ± 1.20

*Difference between groups (P < 0.05).

^ϕ^Acute testosterone (T) treatment with 40 mg intramuscular/animal. Levels of T measured by the time of each intravenous glucose tolerance test (IVGTT).

### Testosterone levels in pregnant sheep and ovariectomized sheep

Treatment with testosterone propionate in the T-mothers was effective in rising plasma levels of testosterone, while control mothers treated with vehicle, showed undetectable levels (Table 2). In ovariectomized sheep, testosterone levels were undetectable by the time of the first IVGTT at 40 weeks of age, while the acute exposure to testosterone was able to increase testosterone levels in both groups of sheep 48 hours after the injection by the time of the second IVGTT (Table 2).

**Table 2:** Summary of testosterone plasma concentrations (ng/ml) in pregnant sheep and ovariectomized sheep during testosterone treatments.

	C-females (n = 6)	T-females (n = 6)
Mothers^a^	ND	2.41 ± 0.94*
Females pre T^b^	ND	ND
Females post T^b^	1.71 ± 0.22	1.72 ± 0.27

*Difference (P < 0.05) between groups within a sample time.

a,b Difference (P < 0.05) between groups in presence or absence of acute testosterone treatment.

^a^Mothers corresponded to pregnant sheep (T-treated) with intramuscular injections of testosterone propionate 30 mg twice per week from day 30 to 90 and 40 mg twice per week from day 91 to 120 of pregnancy. Control dams received the vehicle of the hormone during the same time intervals.

^b^Correspond to control females (C-females) and prenatally exposed to T females (T-females) treated with an acute testosterone injection 48 hours prior to an intravenous glucose tolerance test.

*Difference between groups (P < 0.05).

#### Effects of prenatal exposure to T on insulin secretion and insulin sensitivity in absence of an acute exposure to testosterone. Comparisons between C-females and T-females

**Figure 1:**
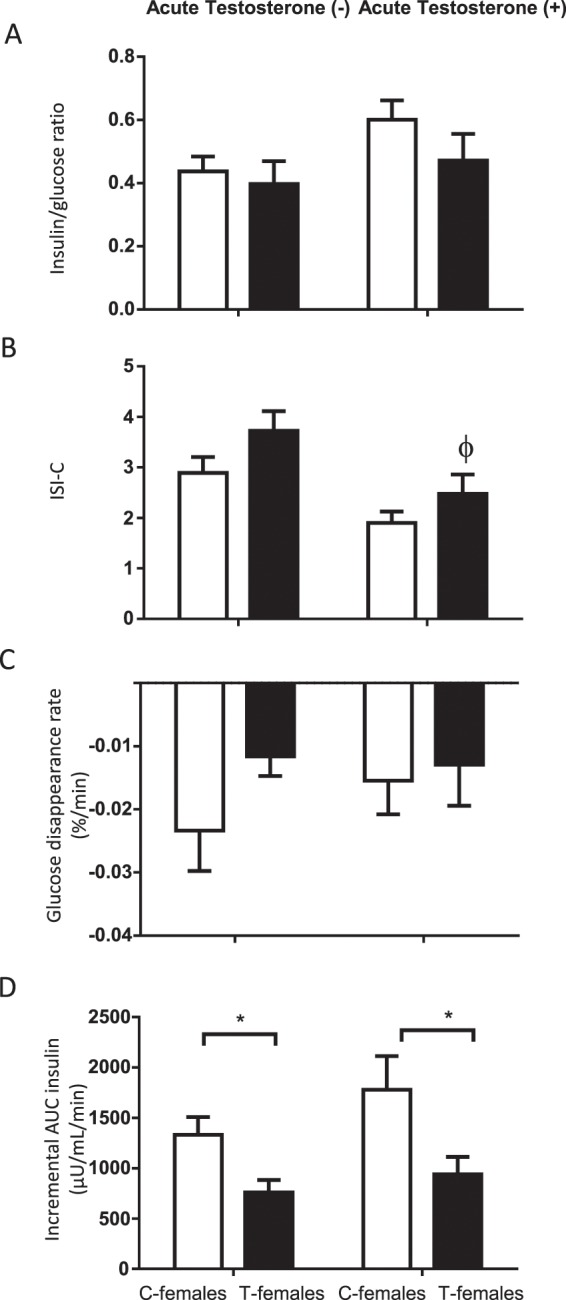
Mean ± SEM end points regarding insulin sensitivity and insulin secretion after an intravenous glucose tolerance test in control females (C-females, open bars, n = 6) and prenatally exposed to testosterone females (T-females, closed bars, n = 6) both before and after an injection of 40 mg of testosterone (Acute testosterone- and acute testosterone+ , respectively). (**A**) Insulin-to-glucose ratio; (**B**) composite insulin sensitivity index (ISI-C); (**C**) glucose disappearance rate; and (**D**) incremental area under the curve of insulin (AUC-I). *P < 0.05 C-females vs. T-females. ^Φ^P < 0.05 T-females pre vs. post acute testosterone.

The insulin-to-glucose ratio, determined in basal samples, as well as the ISI-C and the glucose disappearance rate determined after the first IVGTT, were not significantly different between C- and T-females (Fig. 1 A–C, left side of the graph). However, the incremental AUC of insulin (AUC-I) was significantly lower in T-females (Fig. 1D, left side of the graph).

**Figure 2:**
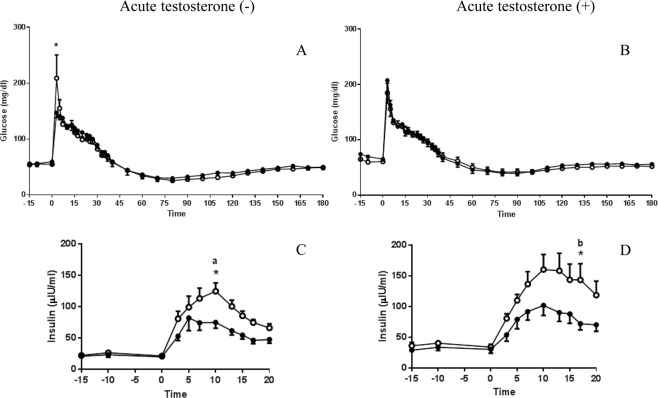
Mean ± SEM glucose concentrations (**A**,**B**) and insulin concentrations ((**C**,**D**)) during and the first 20 minutes after intravenous glucose tolerance test (IVGTT), respectively, in control female sheep (C-females, open circles, n = 6) and prenatally exposed to testosterone female sheep (T-females, closed circles, n = 6) before (**A**,**C**) and after (**B**,**D**) an acute injection of 40 mg of testosterone. Time 0 represents the mean of basal concentrations at -15, -10 and 0 min before glucose infusion. *P < 0.05 denotes difference between C- and T-females at a given time point after glucose infusion. Different letter indicates significant difference within a same group either with or without the acute testosterone treatment.

Glucose concentrations increased significantly 3 minutes after glucose infusion (Fig. 2) and remained high during the first 20 minutes of the IVGTT in both groups (P < 0.05, Time factor F_(8,90)_ = 15.03). A difference in glucose levels between groups was detected at 3 minutes after glucose administration by means of a multiple comparison test (Fig. 2, panel A). Insulin levels increased significantly at 3 minutes post glucose infusion and remained high for the first 20 minutes in C-females, whereas in T-females the increase started at 5 minutes and remained high until minute 13, indicating an effect of time (P < 0.0001, F_(8,90)_ = 10.24) and of prenatal treatment (P < 0.0001, F_(1,90)_ = 32.07). A delay in the peak of insulin was observed in both groups. At 10 min after of the glucose infusion, T-females showed lower insulin concentrations (Fig. 2, panel C).

#### Effect of an acute exposure to testosterone on insulin secretion and insulin sensitivity without prenatal exposure to testosterone. Analysis in C- female sheep

None of the indices of insulin sensitivity or of insulin secretion were significantly changed by the acute treatment with testosterone in C-females (Fig. 1, open bars). In the serial display of glucose and insulin levels it is possible to observe that the levels are similar between the pre and post testosterone situations, except for minute 17 post glucose infusion, where the levels of insulin were higher under testosterone treatment (Fig. 2, panel C and D, open circles).

#### Effect of an acute exposure to testosterone on insulin secretion and insulin sensitivity in prenatally exposure to testosterone. Analysis in T- female sheep

Acute exposure to testosterone caused a significant decrease in ISI-C in T-females (Fig. 1, panel B, closed bars). The AUC-I of insulin, which was already lower due to the prenatal exposure to testosterone, did not change with the acute administration of testosterone in T-females, indicating that insulin secretion by the pancreas was not damage by an acute testosterone treatment. The other parameters of insulin sensitivity were unaltered (Fig. 1, closed bars).

#### Effect of prenatal exposure to testosterone on insulin secretion and insulin sensitivity after testosterone administration. Comparisons between C- and T-females

Similar to the analysis between both groups in absence of an acute exposure to testosterone, T-females showed a significantly lower AUC-I, which indicates that, despite hyperandrogenemia, T-females continue showing a lower secretion of insulin, therefore, the prenatal exposure to testosterone has a major impact on reducing insulin secretion (Fig. 1C, right side of the graph).

The serial display of plasma levels of glucose and insulin along the first 20 minutes of the IVGGT shows that glucose levels rose significantly in both groups, exhibiting a similar pattern not affected by the acute testosterone treatment. According to the ANOVA test insulin levels showed a significant effect of time (P < 0.001, F_(8,90)_ = 6.34) and of the prenatal exposure to testosterone (P < 0.001, F_(1,90)_ = 31.45). As observed in the IVGGT without acute T, both groups showed a delayed peak of insulin. At min 17 after glucose, C-females showed a significantly higher level of insulin (Fig. 2).

## Discusion

We determined whether induced hyperandrogenemia in the absence of the ovaries, has an impact on insulin resistance in ewes prenatally exposed to testosterone. We concluded that hyperandrogenemia lowered insulin sensitivity if there was a previous prenatal exposure to testosterone excess, but also that prenatal exposure to testosterone, regardless of the presence of hyperandrogenemia, decreased glucose-induced secretion of insulin which is consistent with pancreatic reprogramming.

### Developmental programming of testosterone on insulin secretion

The endocrine pancreas is susceptible to programming. In particular, secretory functions of the beta pancreatic cell can be targeted during fetal development at various levels^18^. Among PCOS-like models, this is apparently one of the few studies elucidating the role of fetal exposure to testosterone on pancreatic development and metabolic functions. The fact that T-females had a lower incremental AUC of insulin compared to C-females either with or without the acute testosterone treatment, suggest the presence of testosterone programming, manifested as a lower response to a glucose load. Whether this programming involved a reduced sensitivity of the pancreas to glucose or an impaired insulin synthesis, cannot be established in our model. Other animal models of PCOS, using three times the testosterone dose we used in our model, have shown increased numbers of pancreatic beta cells during adulthood^19^, with no significant effects on beta cell number in the fetus. However, testosterone treatment (20 mg), administered directly to the fetus, increased the number of pancreatic beta cells^20^. This direct testosterone infusion into the fetus also increase insulin secretion after a glucose load at 11 months old of age, indicating a fetal programmed hyperinsulinemia, which is likely to be an end result of insulin resistance. We did not see comparable results in our model. Our results, in fact, show metabolic outcomes that seems in opposition to those previously reported, even considering that the glucose challenges were performed at comparable ages (40–42 weeks vs. 11 months). The source of this difference could lie in a lower dose of testosterone given to our animals that could impact in a differential way. The dose of testosterone we use is intended to resemble both the pattern of increase in plasma testosterone and the plasma concentration of testosterone observed in pregnant PCOS women^21,22^, which recreates a more realistic environment on the fetus. In this setting our results are similar to what has been described in human PCOS^23^. Preliminary experiments from our laboratory have shown a decrease in the total area of the Langerhans islets at 120 days of sheep fetal life under this regimen, which could partially explain the decrease in insulin secretion after a glucose load^24^. The fact that T-females weighed less at birth can point out to a possible reduction in the size of the pancreas as well^25^. Liu *et al*.^26^ suggest an increase in systemic oxidative stress after acute exposure to testosterone coupled to a previous injury in beta cells by prenatal exposure to testosterone, mediated by mechanisms involving the androgen receptor. Interestingly, young male rhesus monkeys prenatally exposed to testosterone also exhibit impaired insulin secretion, without necessarily having high levels of circulating androgens, which suggests a programming effect upon the pancreas^27^. Therefore, our results suggest a programmed failure of the pancreatic beta cells due to a prolonged exposure to testosterone during fetal development.

### Effect of androgenemia on insulin resistance

Hyperandrogenemia is a common feature in PCOS and can increase the risk of insulin resistance. In the present study, a brief hyperandrogenemia reduced the ISI-C only in ewes that were prenatally exposed to testosterone, suggesting that hyperandrogenemia may decrease peripheral sensitivity to insulin. There are reports of testosterone administration to ovariectomized female rats causing a clear insulin resistance^28^. Although, testosterone did not significantly affect insulin sensitivity in C-females, negative effects of testosterone on insulin sensitive tissues have been previously reported. Subcutaneous adipocytes isolated from healthy women and treated *in vitro* with testosterone, had delayed glucose uptake, due to a decrease in the insulin induced phosphorylation of PKC, with no comprise of the upstream insulin signaling^29^, suggesting that testosterone can directly induce insulin resistance in adipose tissue. Adipocytes isolated from PCOS women have reduced glucose uptake, suggesting a disturbance in insulin binding and phosphorylation of its receptor^30^ or a lower GLUT4 translocation^31^. Pre-menopausal women with increased androgens have increased insulin resistance demonstrated by the HOMA model, as well as increased insulin secretion after a glucose load^32^. Recent studies shown that testosterone has specific effects on the adipose tissue of pre-menopausal women increasing pro-inflammatory cytokines, which can affect tissue responses to insulin^33^. Similar animal models have shown differential sensitivity to insulin with liver and skeletal muscle being insulin resistant, and an adipose tissue that remains insulin sensitive^34^. The underlying mechanism involved in insulin resistance in women with PCOS remains somewhat elusive^35^. Since in our model the hyperinsulinemia does not coexist with the hyperandrogenemia, as it has been observed in women with PCOS, but the peripheral IR is still present, as in women with PCOS, the impact that fetal programming has on the response to hyperandrogenemia is critical and can suggest that the origin of this syndrome lies in fetal programming and that hyperinsulinemia is more a consequence than a cause of it.

In summary, prenatal exposure to testosterone induced a programming of the capacity of the pancreas to secrete insulin in response to glucose and predisposes the insulin sensitive tissue to a lower response when challenged with higher testosterone concentrations.

## Data Availability

The datasets generated during and/or analyzed during the current study are available from the corresponding author on reasonable request.
